# Unraveling the complexity of faba bean (*Vicia faba* L.) transcriptome to reveal cold-stress-responsive genes using long-read isoform sequencing technology

**DOI:** 10.1038/s41598-021-00506-0

**Published:** 2021-10-26

**Authors:** Jae Il Lyu, Rahul Ramekar, Jung Min Kim, Nguyen Ngoc Hung, Ji Su Seo, Jin-Baek Kim, Ik-Young Choi, Kyong-Cheul Park, Soon-Jae Kwon

**Affiliations:** 1 Advanced Radiation Technology Institute, Korea Atomic Energy Research Institute, Jeongup, 56212 Korea; 2 Department of Agriculture and Life Industry, Kangwon National University, Chuncheon, 24341 Korea; 3 Department of Horticulture, College of Industrial Sciences, Kongju National University, Yesan, Chungnam 32439 Korea

**Keywords:** Agricultural genetics, Sequencing

## Abstract

Faba bean (*Vicia faba* L.), a globally important grain legume providing a stable source of dietary protein, was one of the earliest plant cytogenetic models. However, the lack of draft genome annotations and unclear structural information on mRNA transcripts have impeded its genetic improvement. To address this, we sequenced faba bean leaf transcriptome using the PacBio single-molecule long-read isoform sequencing platform. We identified 28,569 nonredundant unigenes, ranging from 108 to 9669 bp, with a total length of 94.5 Mb. Many unigenes (3597, 12.5%) had 2–20 isoforms, indicating a highly complex transcriptome. Approximately 96.5% of the unigenes matched sequences in public databases. The predicted proteins and transcription factors included *NB-ARC*, *Myb_domain*, *C3H*, *bHLH*, and heat shock proteins, implying that this genome has an abundance of stress resistance genes. To validate our results, we selected WCOR413-15785, DHN2-12403, DHN2-14197, DHN2-14797, COR15-14478, and HVA22-15 unigenes from the ICE-CBF-COR pathway to analyze their expression patterns in cold-treated samples via qRT-PCR. The expression of dehydrin-related genes was induced by cold stress. The assembled data provide the first insights into the deep sequencing of full-length RNA from faba bean at the single-molecule level. This study provides an important foundation to improve gene modeling and protein prediction.

## Introduction

In addition to cereals, grain legumes are economically valuable crops for smallholder farmers. The reasons for cultivating pulses rather than cereals include the fact that they are a rich source of proteins and carbohydrates essential for the human diet, and that there is substantial demand for them as animal feed^1,2^. Similar to other leguminous species, pulses can fix atmospheric nitrogen into biologically useful ammonia, promoting natural soil nitrogen fertilization^3^. Indeed, the increasing demand for organic food should favor legume-based crops in crop rotation with cereals and oilseeds.

Faba bean (*Vicia faba*) is the fourth most widely grown temperate legume and the only edible pulse crop among *Vicia* species^4^. Compared with other legumes, faba bean is distinguished by its high protein content and balanced amino acid profile^5^. Moreover, its low fat content and relatively low endogenous lipoxygenase activity make faba bean less prone to developing off-flavors than soybean and pea, enabling it to be incorporated into daily diets^6^. Faba bean can also adapt to diverse climatic and soil conditions, providing another advantage over other legumes^7–9^. However, similar to other major legumes, the yield of faba bean remains relatively unstable, which has limited its competitiveness as a commercial crop. This inherent yield instability can be attributed to profligate flowering habits that result in fertilized ovule abortion or insufficient pollination.

Faba bean is also susceptible to various biotic and abiotic stresses, contributing to environment-dependent losses^10^. Although winter-type faba beans have higher yield and protein content than spring-type ones, to increase the yield in cool-temperate regions, faba beans are mainly sown as a spring crop because of the insufficient winter hardiness of the available winter-type cultivars^11^. Several studies have revealed the cold/frost tolerance of faba bean^12–14^. However, investigations of the genes conferring cold tolerance have been hampered by the insufficient genomic information and long-read sequencing transcriptome data for faba bean, which has a relatively large genome (approximately 13 Gb distributed over six chromosomes).

Although genetic variability among plant species is quite large, especially within primitive forms and landraces, and significant progress has been made through conventional breeding, accelerated genetic gains through genomics and associated biotechnology-related research have been limited^15^. There are many reasons why new breeding technologies have not been used for faba bean to the same level as for other crop species. For example, the phenotypic and genetic variability of this species is quite large but remains unexplored, and the method for characterizing its germplasm has not been updated^16^. Furthermore, establishing an efficient faba bean regeneration protocol has been challenging, which has restricted the use of cutting-edge biotechnology-based methods^17^. However, one of the most limiting factors is the considerable size of the faba bean genome, with more than 85% of the genome comprising repetitive sequences^18^.

Moreover, the faba bean is a partially allogamous diploid species with six pairs of extremely large chromosomes^19,20^. Accordingly, the biological and computational analyses required have made it difficult to assemble and annotate such a large genome. Despite these issues, the application of next-generation sequencing (NGS) technology has increased the transcriptome data available for faba bean and enriched the genomic resources for this legume^21–23^.

Transcriptome analysis via NGS is relatively inexpensive and provides sufficient data for clarifying transcriptional and post-transcriptional gene regulation, single-nucleotide variations, and transcript rearrangements. Thus, it is useful for nonmodel crops lacking an available reference genome^24^. However, NGS technology is not ideal for identifying full-length splicing isoforms because the generated reads are short and the associated computational analyses are complicated. Full-length splicing isoforms can produce multiple transcripts for most genes; this increases the protein-coding potential of the genome and increases the transcriptome complexity and flexibility^25,26^.

Isoform sequencing (Iso-seq) using SMRT (single-molecule real-time) technology developed by PacBio (Pacific Biosciences of California Inc., Menlo Park, CA, USA) is an alternative approach for generating single sequence reads of a typical gene or gene family without the need for sequence assembly. Unlike other NGS techniques, the long reads generated by Iso-seq can produce complex assemblies with fully or partially closed gaps with minimal structural errors and accurate gene annotations. Hence, Iso-seq has been applied for comprehensive transcriptome analyses of major crops with large and small genomes^27,28^.

In this study, we used the SMRT Iso-seq protocol for long-read sequencing analysis of faba bean. Furthermore, we identified stress-related transcripts and validated the expression of a few genes involved in cold stress responses. We aimed to characterize and functionally annotate the transcripts to produce data useful for improving gene identification. The generated data may help elucidate the mechanism underlying the tolerance of faba bean to cold stress.

## Results

### Overview of the transcriptome sequencing with SMRT analysis

**Figure 1:**
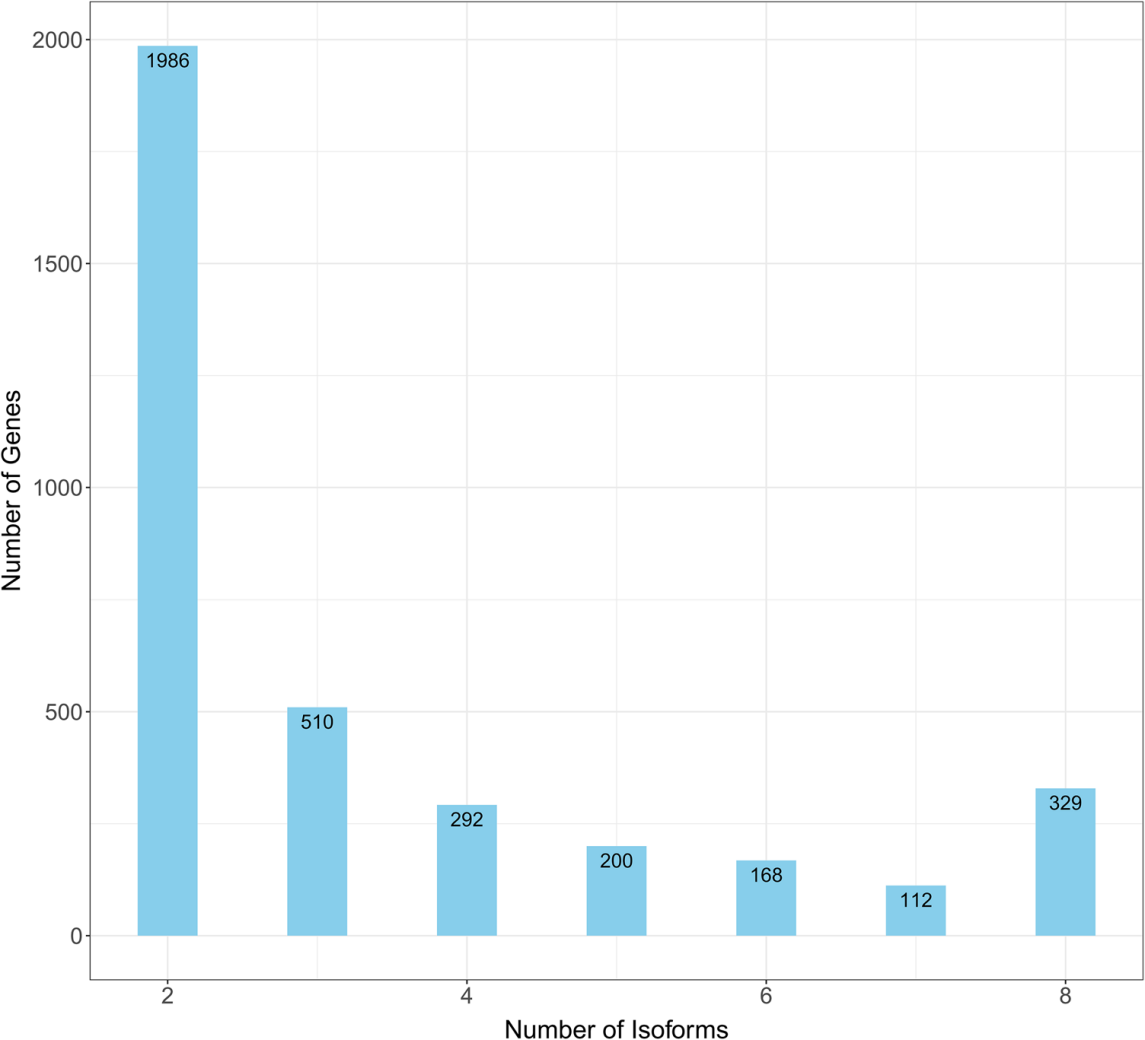
Number of isoforms identified for unigenes in faba bean.

To obtain a full-length representative transcriptome for faba bean, we sequenced and analyzed the total RNA using the PacBio Sequel System. To avoid biases, both long and short transcripts were selected (< 3 kb and ≥ 3 kb). A total of 957,010 polymerase reads (20.1 Gb) were obtained, from which a dataset of clean reads was generated (Supplementary Table S1). Additionally, 35,930,187 subreads were generated, with an average length of 4981 bp and an N_50_ of 5153 bp (Supplementary Table S2). To produce more accurate information, 847,930 circular consensus sequences (CCS) were obtained using SMRTAnalysis 5.0 software (Supplementary Fig. S1). The mean length of the CCS reads was 5542 bp, with an average of 94 passes (Supplementary Table S3). Approximately 89% (754,713) of the reads were classified as full length (containing the 5′ primer, 3′ primer, and poly-A tail), while 730,301 reads were identified as non-full-length non-chimeric (FLNC) reads with few artificial concatemers (Table 1). Finally, redundant transcripts were collapsed using Cogent, after which the Cupcake ToFU pipeline generated 28,569 clustered transcripts (unigenes), with a total length of 94,401,312 bp, and a final gene set comprising 26,321 genes. The sequences in the dataset ranged from 108 to 9666 bp, with an average length of 2786 bp and an N_50_ of 3425 bp (Table 2). Furthermore, we characterized and assigned isoform information to full-length cDNA. We detected alternative splicing in 3597 unigenes with 2–20 isoforms per transcript derived from alternative transcription sites, alternative polyadenylation, or alternative splicing events, with isoforms ranging from 270 to 9666 bp (Fig. 1). The basic workflow for the data analysis is summarized in Supplementary Fig. S2.

**Table 1:** Classification and cluster summary of PacBio SMRT Iso-seq data for faba bean.

Library	1–3 kb	> 3 kb	Total
Reads	309,781	538,149	847,930
Reads with 5′ and 3′ primers	271,156	483,557	754,713
Non-concatamer reads with 5′ and 3′ primers	253,491	477,987	731,478
Non-concatamer reads with 5′ and 3′ primers and Poly-A Tail	253,183	477,118	730,301
Reads without primers	38,625	54,592	93,217
Number of polished, high-quality isoforms	19,609	25,603	45,212
Number of polished low-quality isoforms	42	438	480

**Table 2:** Summary of high-quality non-redundant Iso-seq data for faba bean.

	Collapsing redundant sequence	Collapsing redundant Isoforms
Total number of sequences	33,880	28,569
Total length (bp)	94,401,312	74,743,641
Maximum length	9666	9666
Minimum length	108	108
Total average length	2786	2616

### Functional annotation of faba bean unigenes

**Figure 2:**
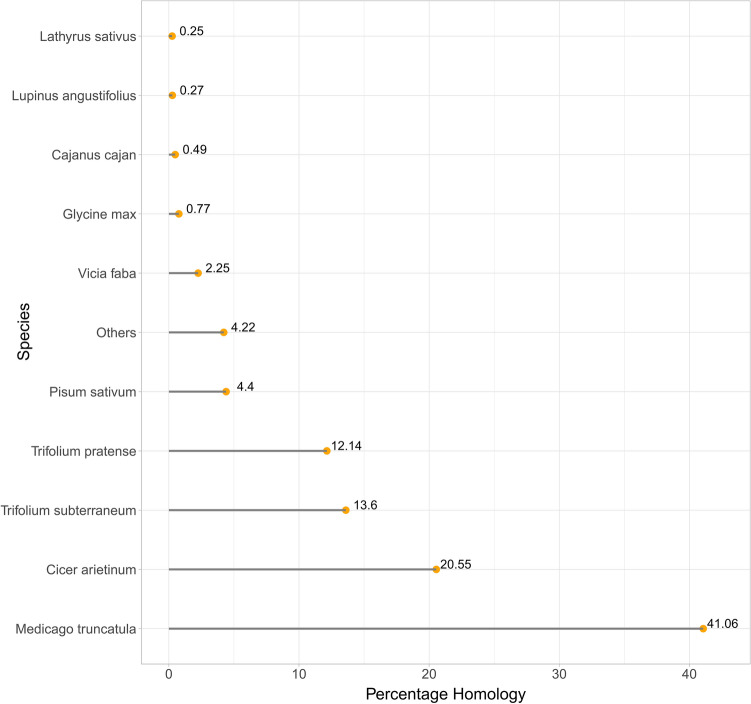
Basic local alignment search tool (BLAST) top-hit species distribution. The substantial similarity to sequences from *Medicago truncatula* and *Cicer arietinum* may reflect a close phylogenetic relationship.

**Figure 3:**
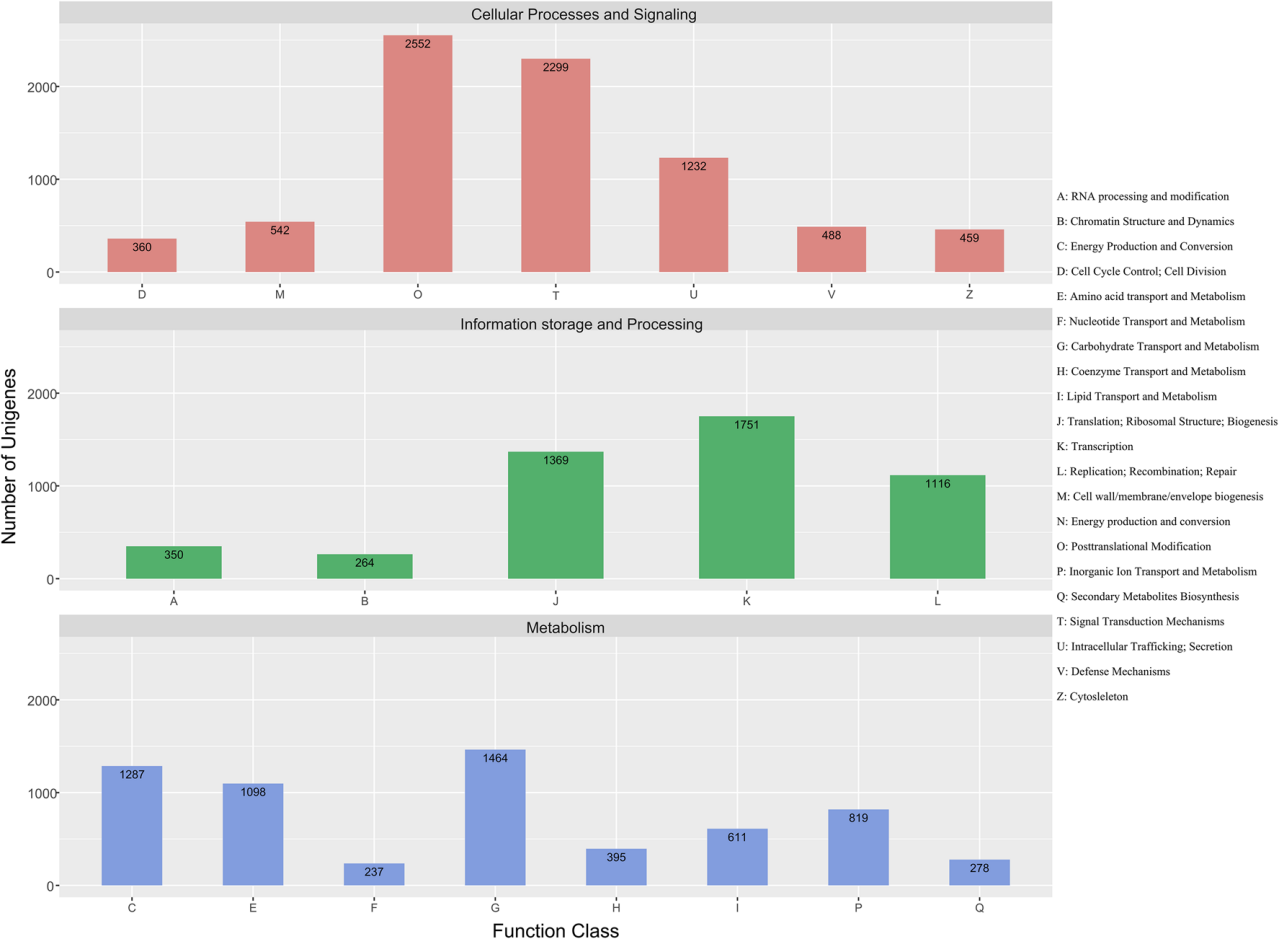
COG functional classification of isoforms.

**Figure 4:**
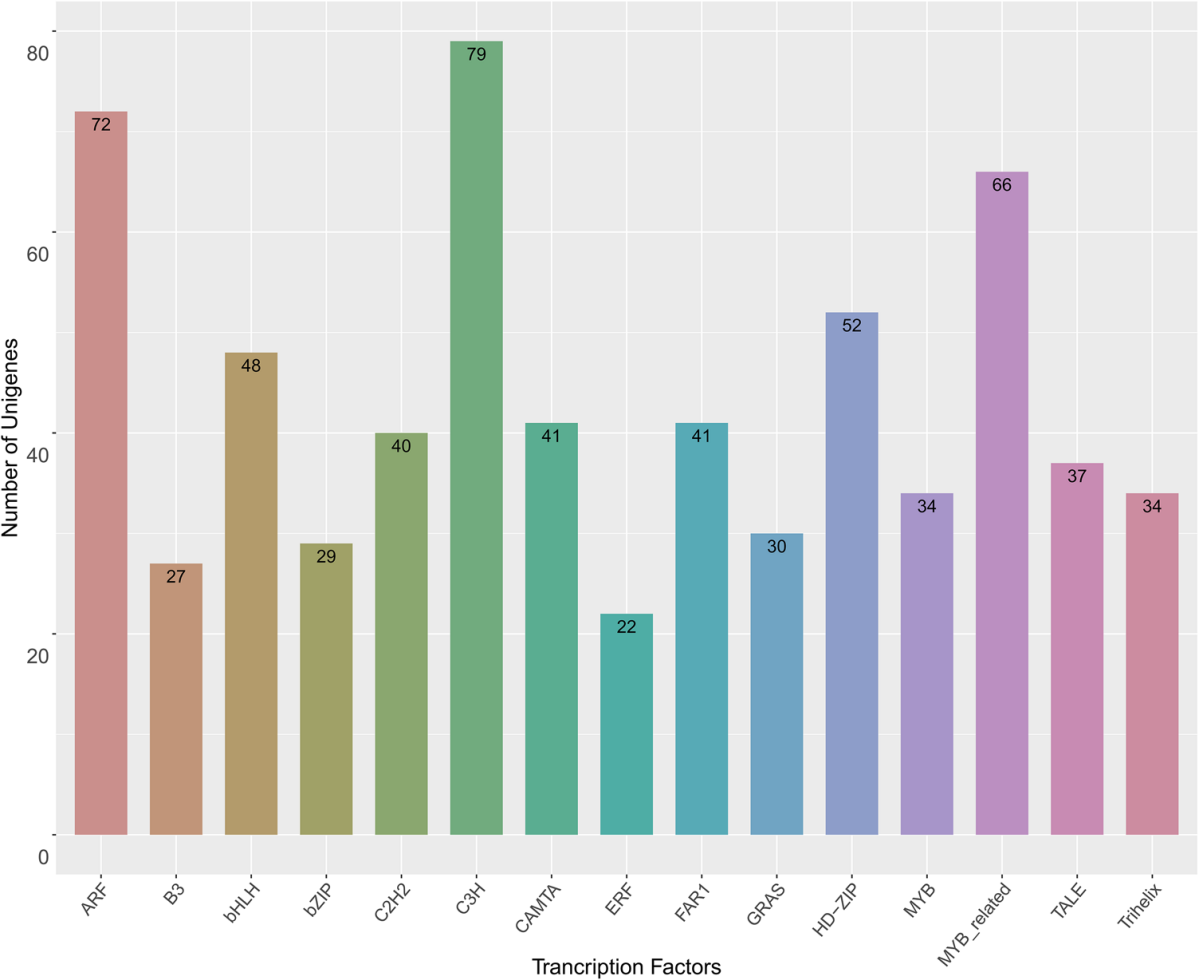
Transcription factors among the isoforms.

To obtain information regarding transcript function, coverage, and quality, we performed a BLASTX analysis^29^. A total of 27,580 unigenes (96.5%) were aligned to sequences in protein databases, including the NR, Pfam, EggNOG, NT, TAIR, and UniProt databases (Table 3). We analyzed homologous species by comparing unigenes to the sequences in the NR database, which revealed that the unigenes mainly matched sequences from *Medicago truncatula* (41.06%), *Cicer arietinum* (20.55%), and *Trifolium pratense* (13.6%) (Fig. 2). To functionally characterize the orthologous proteins, we performed a BLASTX analysis using EggNOG (version 4.5). On the basis of the NOG analysis, 33,635 unigenes were assigned to 25 functional clusters. Excluding the functionally unknown class (14,659 unigenes, 43.58%), “cellular processing and signaling” was the largest class (7937 unigenes), with “post-translational modification” and “signal transduction mechanisms” representing the dominant functional groups. A total of 6189 unigenes (18.40%) belonged to the “metabolism” class and were involved in “energy production and conversion” and “carbohydrate transport and metabolism.” Additionally, 4850 unigenes (14.41%) belonged to the “information storage and processing” class and contributed to “translation, ribosomal structure, and biogenesis” and “transcription” (Fig. 3). The PacBio data were used to predict transcription factors (TFs). A total of 901 putative TFs from 50 families were identified. The major families were associated with C3H-, ARF-, and MYB-related TFs, all of which are considered to regulate metabolism and secondary metabolite biosynthesis in green plants (Fig. 4).

**Table 3:** Annotation of isoforms on the basis of public databases.

Database	Annotation number	300 ≤ length < 1000	Length ≥ 1000 bp
NR	27,811	3131	24,680
UniPort	24,976	2397	22,530
Pfam	25,630	2464	23,137
EggNOG	27,628	2956	24,618
NT	27,580	3017	24,450
TAIR	26,679	2672	23,970
Common	22,891	2097	20,772

**Figure 5:**
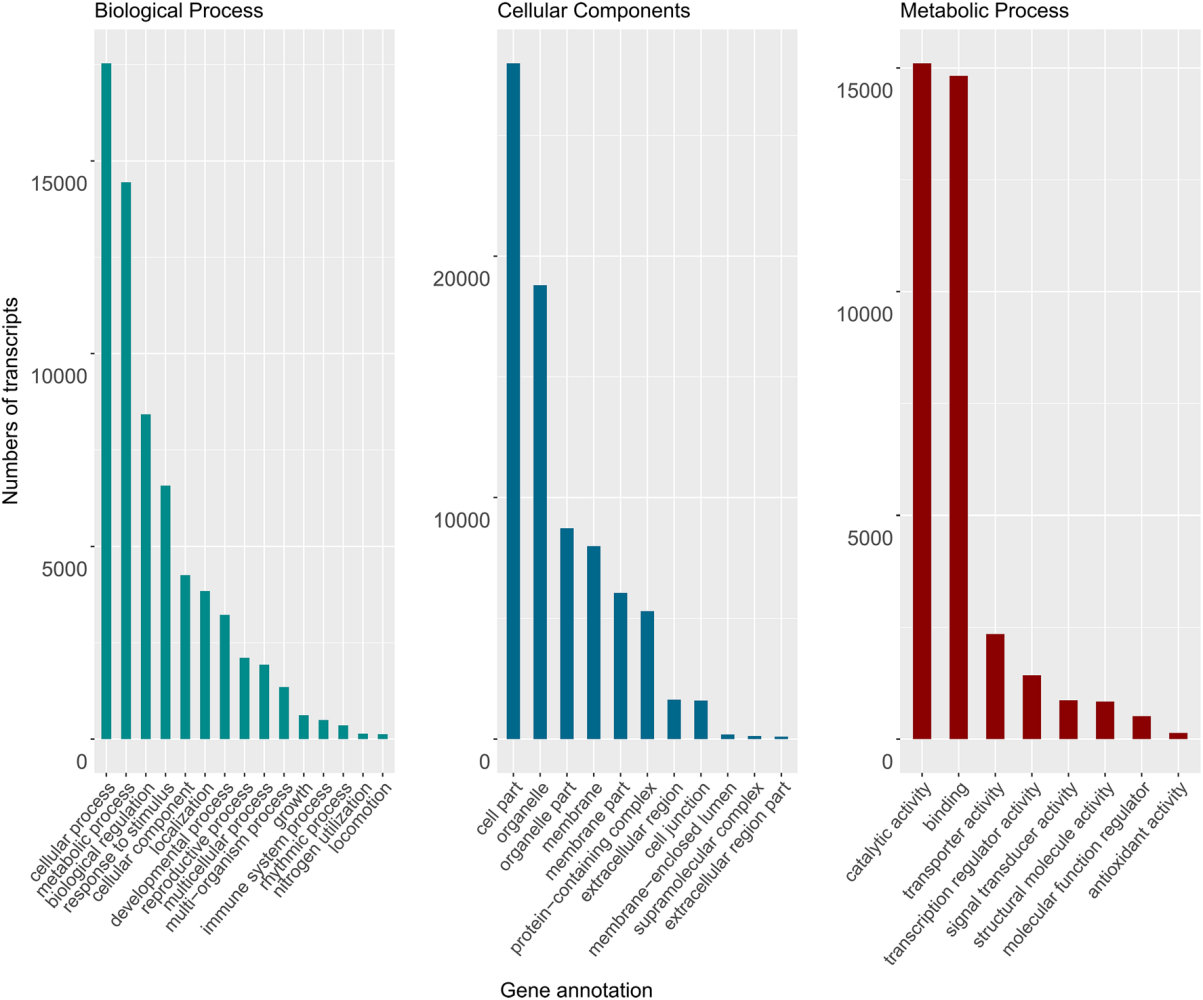
Gene ontology classifications. The results are summarized according to the three main categories: biological process, cellular component, and molecular function. The GO terms assigned to faba bean transcripts with matches in the UniProt database revealed by a BLAST search are presented.

On the basis of the similarity to sequences in the NR database, unigenes were annotated with GO terms. The GO classification assigned unigenes to 62 functional groups in the three main categories as follows: cellular component (12,406, 36.62%), molecular function (9858, 29.1%), and biological process (9844, 29.06%). Approximately 5.25% of the unigenes were not annotated. In the biological process category, “metabolic process” (30.21%) and “cellular process” (14.81%) were the two most represented subcategories. In the molecular function category, the unigenes were primarily associated with “catalytic activity” (42.03%) and “binding” (38.19%), whereas in the cellular component category, the unigenes were mainly related to “cell part” (50.3%) and “organelle” (22.29%) (Fig. 5). The BLASTX results for the faba bean transcriptome (i.e., read length, isoforms, and annotations from all databases) are presented in Supplementary Table S4.

### Identification of stress-related gene families

After screening the Pfam database, 25,630 unigenes were predicted to encode at least one Pfam protein domain (2723 Pfam domains in total). Protein kinase superfamily (1135), protein tyrosine kinase (517), and NB-ARC domain (355) represented most of the protein domains. The other protein families among the 10 largest protein families are listed in Table 4. We also identified genes responsive to biotic and abiotic stresses by searching the Pfam, TAIR, and PlantTFDB databases. In addition to NB-ARC gene family members, we identified 173 heat shock-related genes, 141 Myb DNA-binding domains, and 95 C3H-related domains associated with drought, heat, cold, and salinity stress tolerance (Table 5).

**Table 4:** Top 10 protein family domains encoded by faba bean unigenes revealed by a search of the Pfam database.

No.	Protein family	Unigenes
1	Protein kinase superfamily	1135
2	Protein tyrosine kinase	517
3	NB-ARC domain	355
4	RNA recognition motif	350
5	Chlorophyll A-B binding protein	194
6	ATPase family associated	170
7	Hydrolase	163
8	DEAD (DEAD box helicase)	162
9	KAP (Kinesin-associated protein)	156
10	GTP_EFTU (Elongation factor binding domain)	134

**Table 5:** Representative gene families related to stress resistance in faba bean.

Sr. no.	Gene families	Number of unigenes
1	NB-ARC	355
2	Heat shock protein families	173
3	Myb_DNA binding	141
4	C3H	95
5	ARF	73
6	C2H2	51
7	bHLH	48
8	FAR	41
9	bzip/ABF	29
10	AP2/ERF	28
11	LEA	26
12	WRKY	23
13	COR	20
14	NAC	10

We further classified unigenes associated with the ICE-CBF-COR pathway, a universal pathway related to cold stress responses in plants^30^. These unigenes were related to *ICE1-2*, *CBF1-3*, *DHN2*, *WCOR413*, *COR15a-b*, and *HVA22* (Table 6; Supplementary Table S5). More specifically, 96 unigenes related to the ICE-CBF-COR pathway were detected, with the largest proportion related to *ICE1-2* (45 unigenes), followed by *CBF1-3* (28 unigenes), *WCOR413* (six unigenes), *COR15a-b* and *DHN2* (five unigenes each), and *HAV22* (one unigene). The sequence lengths in the selected gene families ranged from 482 to 3824 bp.

**Table 6:** Isoform information for shortlisted cold tolerance genes.

Unique Isoform ID	Unigene	Number of isoforms	Length
PB.4465.1	WCOR413-15785	1	945
PB.1874.1	DHN2-12403	2	1276
PB.3249.1	DHN2-14197	1	1037
PB.3731.1	DHN2-14797	2	1062
PB.4588.1	HVA22-15951	1	929
PB.3466.1	COR15-14478	2	1072

### Expression analysis of cold-responsive genes

**Figure 6:**
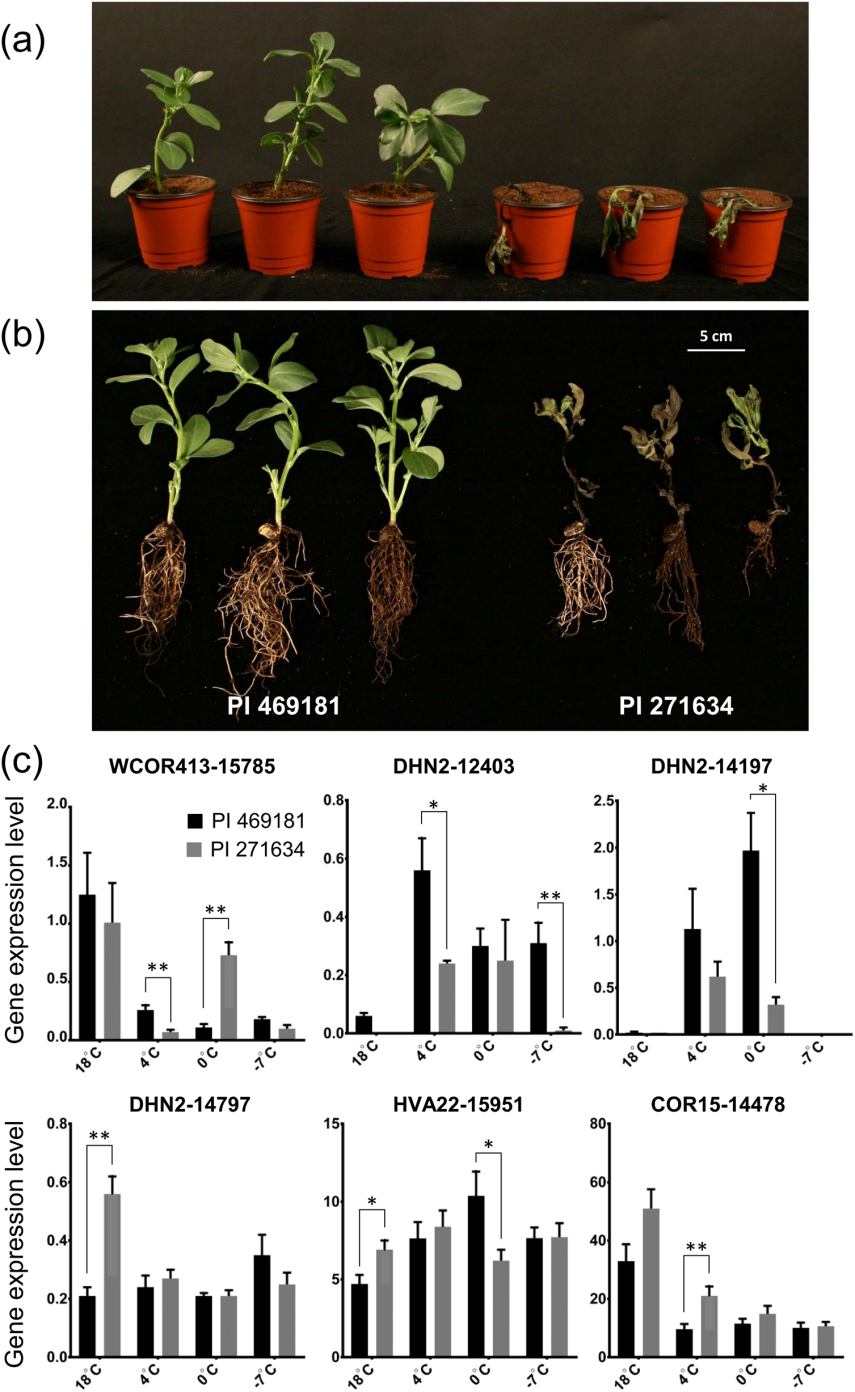
Plant development and expression of cold-related genes in response to cold stress treatment. (**a**) Shoot and (**b**) root development at 4 and − 7 °C. (**c**) Expression levels of candidate cold tolerance genes were determined from the PacBio data. PI 469181 (winter type); PI 271634 (spring type).

To validate the transcriptome data, we analyzed the expression of six unigenes (*WCOR413-15785*, *DHN2-12403*, *DHN2-14197*, *DHN2-14797*, *COR15-14478*, and *HVA22-15*) (primer information—Supplementary Table 6) by qRT-PCR (Fig. 6). In response to cold stress treatment at 4 °C, *DHN2-12403* was the most highly expressed unigene in PI 469181, with an expression level approximately 9.4- and 2.3-fold higher than that under normal conditions (18 °C) and in PI 271634 at 4 °C, respectively. Additionally, during exposure to an extremely low temperature (− 7 °C), the *DHN2-12403* expression level was significantly higher in PI 469181 than in PI 271634 (*P* < 0.01). Similarly, *DHN2-14197* and *HVA22-15951* expression levels were also increased by cold stress conditions (4 and 0 °C) in both accessions, but especially in PI 469181 at 0 °C (*P* < 0.05). In contrast, *WCOR413-15795*, *DHN2-14797*, and *COR15-14478* expression levels were decreased under cold stress conditions, although the expression patterns differed between PI 469181 and PI 271634. These results indicate that *DHN2-12403*, *DHN2-14197*, and *HVA22-15951* are responsive to cold stress. Moreover, the differences in their expression between the cold-tolerant accession (PI 469181) and the cold-susceptible accession (PI 271634) suggest that they may influence the cold tolerance of faba bean. Overall, the reliability of our long-read sequencing data was confirmed by the qRT-PCR analysis.

## Discussion

Most previous studies on the faba bean transcriptome were performed to develop molecular markers or identify stress-related differentially expressed genes, as reviewed elsewhere^31^. A few studies focused on enhancing genomic resources^18,22,32^ and involved short-read sequencing. Although short-read sequencing is useful for quantifying and annotating transcripts in nonmodel plants, it is often unable to accurately capture the entire transcriptome’s true complexity. The faba bean genome has not been annotated, and full-length mRNA sequences with isoform information have been studied by only one research group^33^. Accordingly, we sequenced the faba bean leaf transcriptome using the PacBio SMRT platform to obtain complete transcripts containing untranslated regions and poly-A tails without needing to assemble short reads.

Despite the advantages of long-read sequencing, developing unified and practical approaches to analyzing the data remains a significant challenge. Previous studies applied standard Iso-seq bioinformatic pipelines to generate high-quality isoforms^34,35^. In the current study, we modified the standard pipeline by incorporating the Cogent tool^36^ and the Cupcake ToFU package to refine and reconstruct the coding genome to obtain a comprehensive faba bean transcriptome atlas. Our data included 28,569 high-quality unigenes with a total length of 94 Mb. This genome-wide transcriptome coverage data will be useful for developing molecular markers, studying repetitive elements, and as a reference for quantifying gene expression in future studies. The average length of the faba bean transcripts was 4981 bp, and more than 500 unigenes were longer than 3500 bp, which is substantially longer than the sequences revealed in earlier studies^18,22^. Furthermore, we identified 1349 unigenes with more than 2 and at most 20 isoforms. These results reflect the utility of the SMRT approach for capturing long transcript sequences to enrich the transcriptional information available for large and complex genomes, with implications for functional studies of important genes.

A total of 27,580 unigenes (96.5%) were annotated following an alignment with sequences in six databases. The remaining 989 unigenes lacking significant matches might represent novel genes or correspond to untranslated regions or other noncoding RNAs. BLASTX analyses revealed that more than 40% of the unigenes were significantly similar to sequences from *M. truncatula*, consistent with known phylogenetic relationships^37^ and the results of earlier transcriptome studies^18,22^. The systematic classification of proteins based on NOG analyses suggested that many unigenes were related to post-translational modifications, signal transduction, and transcription. The functional classification according to GO annotations indicated that most of the unigenes assigned to the cellular component category were associated with cell parts and organelles. In the molecular function category, most unigenes were related to catalytic and binding activities, gene regulation, signal transduction, and enzymatically active processes in cells. In the biological process category, unigenes contributing to metabolic and cellular processes were abundant. This functional information may be relevant for identifying genetic networks involved in the regulation of biosynthesis as well as plant growth, development, and stress responses. Overall, the results confirm the accuracy of our sequencing approach and provide the foundation for future molecular research related to genetic gains in faba bean.

The instability of the yield of faba bean is mostly due to exposure to abiotic and/or biotic stresses. Because climate change will likely result in more extreme environmental conditions, enhancing the stress resistance of faba bean has become a high priority. However, stress responses are tightly regulated by complex genetic networks. More specifically, plants have developed sophisticated mechanisms for perceiving subtle changes in the environment and triggering signal transduction to regulate the activity of stress-responsive genes^38^. Transcriptome profiling, especially with long reads, can quickly provide insights into stress-related genetic cascades. Although covering this vast network is beyond the scope of this study, we identified a few important gene families based on Pfam domains and TFs. Many stress-related genes were annotated, including those encoding late embryogenesis abundant **(**LEA) proteins, dehydrins, and heat shock proteins. Earlier research proved that the overexpression of these genes could increase the tolerance to multiple stresses^39^. Most disease resistance proteins contain the NB-ARC domain. In this study, we identified many unigenes encoding this domain.

Unigenes were associated with major TFs, such as MYB, bHLH, and WRKY family members, directly related to stress responses or AB2/ERF, ARF, and C3H TFs, which are in turn related to specific hormone pathways involved in stress responses. These large TF classes are important for regulating gene expression and signal transduction. Overall, these stress resistance genes reflect the diversity of the faba bean transcriptome and may form the basis of future molecular research.

In South Korea, cold stress, including frost, is a major abiotic factor limiting faba bean production. Focusing on cold-responsive genes, we further classified the unigenes associated with the ICE-CBF-COR pathway, including unigenes related to *WCOR413*, *DHN2*, *HAV22*, *CBF1-3*, *ICE1-2*, and *COR15a-b*. Some of these genes encode TFs or positive regulators of TFs, whereas some are cold-regulated genes encoding cryoprotective proteins. For example, the *ICE* gene encodes an MYC-like bHLH transcriptional activator. In response to cold conditions, this activator binds to the *CBF* promoter and activates the expression of downstream cold- and dehydration-responsive genes^41,42^. The *CBF* gene encodes an AP2/ERF (APETALA2/Ethylene-responsive factor)-type TF that specifically binds to the C-repeat dehydration-responsive element to regulate the expression of cold-responsive genes (*COR*)^43^.

In contrast, *COR15a-b*, included in the nuclear genome, encodes a protein belonging to the LEA-4 group with a putative N-terminal signal sequence related to chloroplast import^44^. Freezing stress leads to overexpression of the mature COR15 protein in the chloroplast stroma. This protein functions as a cryoprotectant that stabilizes the membrane bilayer and minimizes electrolyte leakage from cells^42^. Earlier research suggested that COR15 is important for maintaining the activity of protoplast and chloroplast enzymes, such as lactate dehydrogenase, under freezing conditions^45^. Similarly, DHN, a hydrophilic protein with many charged amino acids, belongs to the LEA protein family. Ubiquitous among plant species, DHN proteins accumulate during late embryogenesis, or their production is induced in vegetative tissues following exposure to freezing stress^46^. These proteins protect cell metabolism during cellular dehydration, scavenge hydroxyl radicals, and protect lipid membranes from peroxidation^47^.

We also identified one of the less characterized members of the *COR* gene family, *WCOR413*^48^. Studies proved that the expression of *COR413* genes is strongly induced by cold stress in cereals and *Arabidopsis*^48,49^. To validate our observations, we selected six unigenes encoding COR and dehydrin family members. To examine the expression patterns of these genes, we analyzed two faba bean accessions that vary regarding their tolerance to the cold conditions of South Korean winters^50^. Although the expression patterns of *COR*-related unigenes were generally the same at 4 °C, the expression levels were slightly higher in the cold-tolerant accession, suggesting that these genes contribute to low-temperature stress responses. Their potential roles influencing cold stress tolerance require further investigation.

We revealed a direct correlation between high expression levels and a decrease in temperature regarding the dehydrin-related unigenes. Moreover, we detected an over-accumulation of dehydrin proteins as the temperature decreased, suggesting that they are involved in a late response to freezing or frost stress. The cold tolerance of faba bean is controlled by various genes or quantitative trait loci (QTL)^11^. Previous investigations of faba bean involving SNP and AFLP markers detected QTL associated with frost tolerance in natural accessions and a multi-parent advanced generation inter-cross population^51–53^. In the current study, we identified faba bean isoform sequences and validated the expression of a few cold-responsive genes under cold stress conditions. Thus, to analyze cold tolerance-related genes more thoroughly, additional genetic approaches are needed, including QTL mapping, GWAS, and DEG analyses. We are currently developing faba bean mutant genetic resources produced via gamma irradiation. The isoform data may be relevant to other quality-related loci in faba bean.

In summary, characterizing the full-length faba bean transcriptome should facilitate dissection of the molecular and genetic basis of agronomic traits. Using the data generated in this study, we identified and analyzed transcripts involved in various biological and metabolic processes in faba bean. Combining information regarding the putative stress-related genes and networks with appropriate biotechnological approaches will enable researchers to improve faba bean varieties genetically. The results of this study may be useful for future functional and comparative genomic studies. Moreover, our findings demonstrate the advantages of the SMRT Iso-seq method for identifying genes in nonmodel plants.

## Materials and methods

### Plant materials and RNA extraction

Faba bean accession PI 469181 (highly adapted to the environmental conditions in South Korea) was grown in the experimental field at the Korea Atomic Energy Research Institute (Jeongup, Korea) in 2018. The faba bean accessions were provided by the USDA-National Plant Germplasm System (NPGS) (http://npgsweb.ars-grin.gov/) at Pullman, WA, USA. All experiments were carried out in accordance with national regulations in Korea. Young apical stems and leaves were collected, immediately frozen in liquid nitrogen, and ground into a fine powder. Total RNA was isolated from the leaves using the RiboPure Kit (Applied Biosystems, Foster City, CA, USA). The RNA concentration was determined using the ND1000 spectrophotometer (NanoDrop Technologies, DE, USA), whereas the RNA quality was assessed using the 2100 Bioanalyzer (Agilent Technologies Inc., Santa Clara, CA, USA). Samples comprising 1–10 μg of RNA with an RNA integrity number (RIN) > 8.0 were used for sequencing.

### Library preparation and SMRT sequencing

A total of 6 μg of RNA was used to synthesize cDNA using the Clontech SMARTer PCR cDNA Synthesis Kit (Takara Bio USA Inc., CA, USA). Large double-stranded cDNA was produced in optimized PCR cycles, after which the BluePippin™ Size Selection System (Sage Science Inc., Beverly, MA, USA) was used for size fractionation and selection (1–10 kb). A template library was prepared using the SMRTbell library kit (Pacific Biosciences Inc., CA, USA) and the PacBio RSII platform, in accordance with the manufacturer’s protocol at the National Instrumentation Center for Educational Management (Seoul National University, Seoul, South Korea). Libraries were extracted in two batches (< 3 kb and > 3 kb) using the BluePippin™ Size Selection System. Two SMRTbell libraries were constructed with the Pacific Biosciences DNA Template Prep Kit (version 2.0) for SMRT sequencing on the Pacific Biosciences Sequel System.

### Quality filtering and long-read processing

The PacBio long raw reads were processed using SMRTlink (version 4.0) software. The pipeline included generating CCS, identifying full-length reads (classification), clustering isoforms, and polishing to generate high-quality isoforms. The obtained subread BAM file reads were processed into error-corrected CCS. After identifying the 5′ and 3′ adapters and the poly-A tail, CCS were classified into full-length and non-full-length reads. The CCS with all 5′ and 3′ reads were designated as non-concatemer reads with 5′ and 3′ primers, whereas those with all three elements and no additional copies of the adapter sequence within the DNA fragment were designated as non-concatemer reads with 5′ and 3′ primers and a poly-A tail or full-length non-concatemer (FLNC) reads. Consensus isoforms were identified from the FLNC reads using the ICE (Iterative Clustering for Error Correction) algorithm and were further polished based on non-full-length reads using Quiver to obtain high-quality isoforms with post-correction accuracy exceeding 99%^54^. Redundant sequences were collapsed using the COding GENome reconstruction tool (Cogent version 3.9)^36^ and unique isoforms were defined using the Cupcake ToFU package (https://github.com/Magdoll/cDNA_Cupcake)^55^.

### Functional annotation and classification

Functional annotations were completed using BLASTX (E-value cut-off of 10^−6^) to screen the following databases: NR (NCBI non-redundant proteins), NT (nucleotide database), UniProt, EggNOG (orthology prediction), GO (gene ontology), Pfam (conserved protein families and domains), and TAIR (*Arabidopsis* information resources). On the basis of the annotations, TFs) in PlantTFDB 4.0 were identified^56^.

### Cold stress treatments

We identified a set of gene families involved in abiotic and biotic stress responses based on the protein domains. We subsequently identified six gene groups, WCOR413 (wheat COR-413), DHN2 (dehydrin), HVA22, CBF1 (C-repeat binding factor), ICE1-2 (inducer of CBF expression), and COR15a-b (cold regulated 15), involved in the ICE-CBF-COR pathway associated with cold stress responses in diverse crops. We randomly selected six unigenes from these groups and studied their expression profiles to validate the transcriptome data. To confirm the cold tolerance/susceptibility phenotype of the two accessions and validate the identification of cold-responsive genes, we conducted cold stress experiments as previously described^51^, with some modifications (e.g., exposure times and the lowest temperature). Briefly, the cold-tolerant line (PI 469181) and the cold-susceptible line (PI 271634) were grown in pots, which were placed in a growth chamber (Vision Science, Korea) set at 18 °C for 2 weeks. The temperature in the growth chamber was gradually lowered at a rate of 1 °C/h and then held at 4 °C for 3 days (12-h light/12-h dark). To investigate the gene expression levels at extremely low temperatures, the temperature was lowered further (1 °C/h) to − 7 °C. After 12 h of exposure to − 7 °C, the plants were allowed to recover at 18 °C.

### Quantitative real-time (qRT)-PCR

Total RNA was isolated from cold-treated/untreated leaf tissue using TRIzol reagent and treated with DNase I (Ambion, USA). For both accessions, RNA samples were prepared in triplicate. The extracted RNA (1 μg) served as a template to synthesize the first-strand cDNA using the SuperScript III First-Strand Synthesis SuperMix (Invitrogen, USA). Gene-specific primers were designed for the selected unigene sequences using Primer3 software (http://primer3plus.com/primer3web/primer3web_input.htm). A qRT-PCR assay was performed using the iTaq Universal SYBR Green SuperMix (Bio-Rad, USA) and the Bio-Rad CFX96 real-time PCR detection system. The PCR program was as follows: 94 °C for 10 min; and then 45 cycles of 94 °C for 10 s, 60 °C for 15 s, and 72 °C for 30 s. The analysis was completed using three independent biological replicates. Gene expression levels were normalized against the expression of the cyclophilin (*CYP2*) gene^57^ and calculated according to the 2^−ΔΔCt^ comparative threshold method^58^.

## Supplementary Information

Supplementary Information 1.

Supplementary Information 2.

## Data Availability

The PacBio sequencing raw reads can be accessed from the NCBI Sequence Read Archive (SRA) under accession number SRX10148269.
