# Supplementary material for: Unraveling the complexity of faba bean (Vicia faba L.) transcriptome to reveal cold-stress-responsive genes using long-read isoform sequencing technology
Source: Sci Rep. 2021 Oct 26;11:21094. doi: 10.1038/s41598-021-00506-0 (PMC8548339; doi:10.1038/s41598-021-00506-0)
Supplement: Supplementary file 1 — Supplementary Information 1. [file 41598_2021_506_MOESM1_ESM.pdf]

Supplementary Table 1. Summary of the polymerase read statistics for Fababeans from PacBio sequencing

| Library | Polymerase Read length (bp) | Polymerase Reads (bp) | Polymerase Read N50 (bp) | Polymerase Read length (bp) |
|---|---|---|---|---|
| 1-3kb | 31819542182 | 330824 | 167709 | 96183 |
| >3kb | 47391599705 | 626186 | 141667 | 75683 |
| Total | 79211141887 | 957010 | 309376 | 171866 |

Supplementary Table 2. Summary of the Subread statistics for Fababeans from PacBio

| Library | Subread length (bp) | Subreads | Subread N50 (bp) | Avg. Subread length (bp) |
|---|---|---|---|---|
| 1-3kb | 30808203355 | 23086802 | 1554 | 1334 |
| >3kb | 46843019830 | 12843385 | 3599 | 3647 |
| Total | 77651223185 | 35930187 | 5153 | 4981 |

sequencing

Supplementary Table 3. Summary of circular consensus sequence (CCS)

| Category | 1-3kb | >3kb | Total |
|---|---|---|---|
| CCS reads | 3,09,781 | 5,38,149 | 8,47,930 |
| Number of CCS bases | 52,71,42,715 | 2,06,74,90,009 | 2,59,46,32,724 |
| CCS Read Length (mean) | 1,701 | 3,841 | 5,542 |
| Number of Passes (mean) | 72 | 22 | 94 |

Supplementary Table 5. Summary of gene families involved cold stress

a) List of WCOR413 genes selected from the Fababean full-length gene set

| No. | Contig | NR | Pfam | NT |
|---|---|---|---|---|
| 1 | FaBabeanB_3kb_HQ_transcript/15469 | cold-regulated 413 plasma membrane protein 1 [Cajanus cajan] | PF05562.8 | Medicago truncatula cold acclimation protein WCOR413 mRNA |
| 2 | FaBabeanB_3kb_HQ_transcript/15785 | cold-regulated 413 inner membrane protein 1, chloroplastic [Medicago truncatula] | PF05562.8 | Medicago truncatula cold acclimation protein WCOR413 mRNA |
| 3 | FaBabeanB_3kb_HQ_transcript/16168 | cold-regulated 413 inner membrane protein 1, chloroplastic [Medicago truncatula] | PF05562.8 | Medicago truncatula cold acclimation protein WCOR413 mRNA |
| 4 | FaBabeanB_3kb_HQ_transcript/16543 | cold-regulated 413 plasma membrane protein 1 [Cajanus cajan] | PF05562.8 | Medicago truncatula clone JCVI-FLMt-11I1 unknown mRNA |
| 5 | FaBabeanB_3kb_HQ_transcript/16601 | cold-regulated 413 plasma membrane protein 1 [Cajanus cajan] | PF05562.8 | Medicago truncatula cold acclimation protein WCOR413 mRNA |
| 6 | FaBabeanB_3kb_HQ_transcript/17310 | cold-regulated 413 plasma membrane protein 1 [Cajanus cajan] | PF05562.8 | Medicago truncatula clone JCVI-FLMt-11I1 unknown mRNA |

b) List of DHN2 genes selected from the Fababean full-length gene set.

| No. | Contig | NR | Pfam | NT |
|---|---|---|---|---|
| 1 | FaBabeanB_3kb_HQ_transcript/12403 | dehydrin-cognate [Pisum sativum] | PF00257 | P. sativum dhn-cog gene encoding dehydrin-cognate |
| 2 | FaBabeanB_3kb_HQ_transcript/14108 | dehydrin-cognate [Pisum sativum] | PF00257 | P. sativum dhn-cog gene encoding dehydrin-cognate |
| 3 | FaBabeanB_3kb_HQ_transcript/14197 | dehydrin-cognate [Pisum sativum] | PF00257 | P. sativum dhn-cog gene encoding dehydrin-cognate |
| 4 | FaBabeanB_3kb_HQ_transcript/14797 | dehydrin-cognate [Pisum sativum] | PF00257 | P. sativum dhn-cog gene encoding dehydrin-cognate |
| 5 | FaBabeanB_3kb_HQ_transcript/16189 | dehydrin-cognate [Pisum sativum] | PF00257 | P. sativum dhn-cog gene encoding dehydrin-cognate |

c) List of CBF genes selected from the Fababean full-length gene set.

| | Contig | NR | Pfam | NT |
|---|---|---|---|---|
| 1 | FaBabeanB_3kb_HQ_transcript/10266 | ethylene-responsive element binding protein [Medicago falcata] | K4BN49.1 PF00847.17;AP2; | Medicago truncatula ethylene response factor mRNA |
| 2 | FaBabeanB_3kb_HQ_transcript/11169 | putative ethylene responsive factor [Vicia faba] | I1KYC2.1 PF00847.17;AP2; | Pisum sativum AT-rich element binding factor 2 (ATF2) mRNA, complete cds |
| 3 | FaBabeanB_3kb_HQ_transcript/11742 | ethylene-responsive transcription factor ERF060 [Medicago truncatula] <KEH20859.1 ethylene response factor [Medicago truncatula] | I1K4K5.1 PF00847.17;AP2; | Trifolium repens clone BAC 88n22 myo-inositol-1-phosphate synthase, bristled-like protein, ethylene insensitive-like protein, bZIP transcription factor, acyl-coA oxidase, and dehydration responsive element binding protein genes, complete cds; and unknown genes |
| 4 | FaBabeanB_3kb_HQ_transcript/12219 | putative ethylene responsive factor [Vicia faba] | I1KYC2.1 PF00847.17;AP2; | Pisum sativum AT-rich element binding factor 2 (ATF2) mRNA, complete cds |
| 5 | FaBabeanB_3kb_HQ_transcript/12237 | putative ethylene responsive factor [Vicia faba] | I1KYC2.1 PF00847.17;AP2; | Pisum sativum AT-rich element binding factor 2 (ATF2) mRNA, complete cds |
| 6 | FaBabeanB_3kb_HQ_transcript/13400 | hypothetical protein PHAVU_002G016700g [Phaseolus vulgaris] <ESW28771.1 hypothetical protein PHAVU_002G016700g [Phaseolus vulgaris] | Q8W3M3.1 PF00847.17;AP2; | Medicago truncatula chromosome 5 clone mth2-28f11, COMPLETE SEQUENCE |
| 7 | FaBabeanB_3kb_HQ_transcript/14307 | ethylene-responsive transcription factor-like protein At4g13040 isoform X2 [Medicago truncatula] <KEH22585.1 ethylene response factor [Medicago truncatula] | K4CTN5.1 PF00847.17;AP2; | Medicago truncatula ethylene response factor mRNA |

| | | | | |
|---|---|---|---|---|
| 8 | FaBabeanB_3kb_HQ_transcript/14546 | putative ethylene responsive factor [Vicia faba] | I1KYC2.1 PF00847.17;AP2; | Pisum sativum AT-rich element binding factor 2 (ATF2) mRNA, complete cds |
| 9 | FaBabeanB_3kb_HQ_transcript/14633 | hypothetical protein PHAVU_002G016700g [Phaseolus vulgaris] <ESW28771.1 hypothetical protein PHAVU_002G016700g [Phaseolus vulgaris] | Q8W3M3.1 PF00847.17;AP2; | Medicago truncatula ethylene response factor mRNA |
| 10 | FaBabeanB_3kb_HQ_transcript/14636 | unknown [Medicago truncatula] | A0A072V5S6.1 PF00847.17;AP2; | Vicia faba putative ethylene responsive factor (ERF2) mRNA, complete cds |
| 11 | FaBabeanB_3kb_HQ_transcript/14775 | putative ethylene responsive factor [Vicia faba] | I1KYC2.1 PF00847.17;AP2; | Pisum sativum AT-rich element binding factor 2 (ATF2) mRNA, complete cds |
| 12 | FaBabeanB_3kb_HQ_transcript/15647 | ethylene-responsive transcription factor ERF104 [Medicago truncatula] <KEH41321.1 ethylene response factor [Medicago truncatula] | A0A072VI96.1 PF00847.17;AP2; | Medicago truncatula ethylene response factor mRNA |
| 13 | FaBabeanB_3kb_HQ_transcript/15870 | DREB transcription factor 1 [Medicago ruthenica] | A0A072UL75.1 PF00847.17;AP2; | Medicago sativa subsp. falcata putative DRE-binding protein DREB1 (DREB1) mRNA, complete cds |
| 14 | FaBabeanB_3kb_HQ_transcript/15890 | ethylene response factor 8 [Medicago sativa] | I1LCJ0.1 PF00847.17;AP2; | Medicago sativa ethylene response factor 8 (ERF8) mRNA, complete cds |
| 15 | FaBabeanB_3kb_HQ_transcript/16889 | ethylene-responsive transcription factor rap2-1-like protein [Trifolium pratense] | M1ADM5.1 PF00847.17;AP2; | PREDICTED: Cicer arietinum ethylene-responsive transcription factor RAP2-1-like (LOC101512420), mRNA |
| 16 | FaBabeanB_3kb_HQ_transcript/17432 | pathogenesis-related genes transcriptional activator PTI5 [Medicago truncatula] <AES63941.1 ethylene response factor [Medicago truncatula] | K7M1F8.1 PF00847.17;AP2; | Medicago truncatula ethylene response factor mRNA |

| | | | | |
|---|---|---|---|---|
| 17 | FaBabeanB_3kb_HQ_transcript/4409 | APETAL2-like protein [Pisum sativum] | A0A060CZG0.1 PF00847.17;AP2; | Pisum sativum APETAL2-like protein mRNA, complete cds |
| 18 | FaBabeanB_3kb_HQ_transcript/4589 | PREDICTED: floral homeotic protein APETALA 2-like [Cicer arietinum] | A0A060CZG0.1 PF00847.17;AP2; | Pisum sativum APETAL2-like protein mRNA, complete cds |
| 19 | FaBabeanB_3kb_HQ_transcript/5672 | floral homeotic protein APETALA 2 isoform X2 [Medicago truncatula] <AES94650.1 AP2 domain transcription factor [Medicago truncatula] | A0A060CZG0.1 PF00847.17;AP2; | Medicago truncatula AP2 domain transcription factor mRNA |
| 20 | FaBabeanB_3kb_HQ_transcript/6011 | ethylene-responsive transcription factor erf118-like protein [Trifolium pratense] | K4BXB9.1 PF00847.17;AP2; | Medicago truncatula ethylene-responsive transcription factor ERF118-like protein mRNA |
| 21 | FaBabeanB_3kb_HQ_transcript/6590 | ethylene-responsive transcription factor rap2-7-like protein [Trifolium pratense] | A0A060CZG0.1 PF00847.17;AP2; | Medicago truncatula AP2-like ethylene-responsive transcription factor mRNA |
| 22 | FaBabeanB_3kb_HQ_transcript/6834 | ethylene-responsive transcription factor RAP2-4 [Medicago truncatula] <AEX93413.1 putative AP2/EREBP transcription factor [Medicago truncatula] <KEH34901.1 ethylene response factor [Medicago truncatula] | C5YKT7.1 PF00847.17;AP2; | Medicago truncatula ethylene response factor mRNA |
| 23 | FaBabeanB_3kb_HQ_transcript/7404 | ethylene-responsive transcription factor erf118-like protein [Trifolium pratense] | K4BXB9.1 PF00847.17;AP2; | Medicago truncatula ethylene-responsive transcription factor ERF118-like protein mRNA |
| 24 | FaBabeanB_3kb_HQ_transcript/7813 | ethylene-responsive element binding protein [Medicago falcata] | K4BN49.1 PF00847.17;AP2; | Medicago truncatula ethylene response factor mRNA |
| 25 | FaBabeanB_3kb_HQ_transcript/8585 | ethylene-responsive element binding protein [Medicago falcata] | K4BN49.1 PF00847.17;AP2; | Medicago truncatula ethylene response factor mRNA |

| | | | | |
|---|---|---|---|---|
| 26 | FaBabeanB_3kb_HQ_transcript/9776 | ethylene-responsive element binding protein [Medicago falcata] | K4BN49.1 PF00847.17;AP2; | Medicago truncatula ethylene response factor mRNA |
| 27 | FaBabeanB_3kbup_HQ_transcript/14070 | disease resistance protein rga3-like [Trifolium pratense] | I1JHM5.1 PF00847.17;AP2; | PREDICTED: Cicer arietinum disease resistance protein RGA2-like (LOC101498365), mRNA |
| 28 | FaBabeanB_3kbup_HQ_transcript/22496 | ethylene-responsive transcription factor rap2-7-like protein [Trifolium pratense] | A0A060CZG0.1 PF00847.17;AP2; | PREDICTED: Cicer arietinum ethylene-responsive transcription factor RAP2-7 (LOC101489539), mRNA |
| 29 | FaBabeanB_3kbup_HQ_transcript/8966 | disease resistance protein rga3-like [Trifolium pratense] | I1JHM5.1 PF00847.17;AP2; | Medicago truncatula clone mth2-24f15, complete sequence |
| 30 | FaBabeanB_3kbup_HQ_transcript/9360 | disease resistance protein rga3-like [Trifolium pratense] | I1JHM5.1 PF00847.17;AP2; | PREDICTED: Cicer arietinum disease resistance protein RGA2-like (LOC101498365), mRNA |

d) List of ICE genes selected from the Fababean full-length gene set.

| | Contig | NR | Pfam | NT |
|---|---|---|---|---|
| 1 | FaBabeanB_3kb_HQ_transcript/10596 | transcription factor bHLH63 isoform X2 [Medicago truncatula] <AES74186.2 BHLH transcription factor [Medicago truncatula] | M4D4Y3.1 PF00010.23;HLH; | Medicago truncatula clone JCVI-FLMt-18N21 unknown mRNA |
| 2 | FaBabeanB_3kb_HQ_transcript/10652 | hypothetical protein TSUD_333600 [Trifolium subterraneum] | G7ICN0.1 PF00010.23;HLH; | PREDICTED: Cicer arietinum transcription factor UNE12 (LOC101502533), transcript variant X2, mRNA |
| 3 | FaBabeanB_3kb_HQ_transcript/10870 | transcription factor bHLH63 [Medicago truncatula] <AES89129.1 transcription factor [Medicago truncatula] | M4D4Y3.1 PF00010.23;HLH; | Medicago truncatula transcription factor mRNA |
| 4 | FaBabeanB_3kb_HQ_transcript/11233 | transcription factor bHLH63 [Medicago truncatula] <AES89129.1 transcription factor [Medicago truncatula] | M4D4Y3.1 PF00010.23;HLH; | Medicago truncatula transcription factor mRNA |
| 5 | FaBabeanB_3kb_HQ_transcript/11366 | transcription factor bHLH63 [Medicago truncatula] <AES89129.1 transcription factor [Medicago truncatula] | M4D4Y3.1 PF00010.23;HLH; | Medicago truncatula transcription factor mRNA |
| 6 | FaBabeanB_3kb_HQ_transcript/11633 | transcription factor bHLH7 isoform X1 [Medicago truncatula] <AES60927.1 helix loop helix DNA-binding domain protein [Medicago truncatula] | A0A072VLC0.1 PF00010.23;HLH; | Medicago truncatula helix loop helix DNA-binding domain protein mRNA |
| 7 | FaBabeanB_3kb_HQ_transcript/11828 | transcription factor bHLH63 [Medicago truncatula] <AES89129.1 transcription factor [Medicago truncatula] | M4D4Y3.1 PF00010.23;HLH; | Medicago truncatula transcription factor mRNA |
| 8 | FaBabeanB_3kb_HQ_transcript/11855 | transcription factor BIM2 [Medicago truncatula] <AES97297.1 transcription factor BIM2-like protein [Medicago truncatula] | G7JZB3.1 PF00010.23;HLH; | PREDICTED: Cicer arietinum transcription factor BIM2 (LOC101511604), mRNA |
| 9 | FaBabeanB_3kb_HQ_transcript/11858 | hypothetical protein TSUD_141490 [Trifolium subterraneum] | I1GN25.1 PF00010.23;HLH; | Medicago truncatula BHLH transcription factor-like protein mRNA |
| 10 | FaBabeanB_3kb_HQ_transcript/11871 | transcription factor BIM2 [Medicago truncatula] <AES97297.1 transcription factor BIM2-like protein [Medicago truncatula] | G7JZB3.1 PF00010.23;HLH; | PREDICTED: Cicer arietinum transcription factor BIM2 (LOC101511604), mRNA |
| 11 | FaBabeanB_3kb_HQ_transcript/12406 | hypothetical protein TSUD_00230 [Trifolium subterraneum] | B9IAD1.1 PF00010.23;HLH; | PREDICTED: Cicer arietinum transcription factor bHLH128-like (LOC101501688), mRNA |
| 12 | FaBabeanB_3kb_HQ_transcript/12832 | PREDICTED: transcription factor bHLH79-like [Cicer arietinum] | K7K628.1 PF00010.23;HLH; | PREDICTED: Cicer arietinum transcription factor bHLH79-like (LOC101513654), mRNA |
| 13 | FaBabeanB_3kb_HQ_transcript/13571 | PREDICTED: transcription factor ILR3-like [Cicer arietinum] | B9SA09.1 PF00010.23;HLH; | Medicago truncatula BHLH domain class transcription factor mRNA |
| 14 | FaBabeanB_3kb_HQ_transcript/15326 | hypothetical protein TSUD_172970 [Trifolium subterraneum] | B9IBW9.2 PF00010.23;HLH; | PREDICTED: Cicer arietinum transcription factor UNE10 (LOC101492173), mRNA |
| 15 | FaBabeanB_3kb_HQ_transcript/15388 | transcription factor bHLH47-like protein [Trifolium pratense] | M0SG68.1 PF00010.23;HLH; | PREDICTED: Cicer arietinum transcription factor bHLH47 (LOC101512450), mRNA |

| | | | | |
|---|---|---|---|---|
| 16 | FaBabeanB_3kb_HQ_transcript/15669 | hypothetical protein TSUD_101990 [Trifolium subterraneum] | B9SA09.1 PF00010.23;HLH; | Medicago truncatula BHLH domain class transcription factor mRNA |
| 17 | FaBabeanB_3kb_HQ_transcript/15835 | hypothetical protein TSUD_101990 [Trifolium subterraneum] | B9SA09.1 PF00010.23;HLH; | Medicago truncatula BHLH domain class transcription factor mRNA |
| 18 | FaBabeanB_3kb_HQ_transcript/16148 | PREDICTED: transcription factor bHLH104-like [Cicer arietinum] | G7I5E7.1 PF00010.23;HLH; | Medicago truncatula BHLH domain class transcription factor mRNA |
| 19 | FaBabeanB_3kb_HQ_transcript/2201 | hypothetical protein TSUD_208340 [Trifolium subterraneum] | A0A0D3CAD2.1 PF00010.23;HLH; | Medicago truncatula transcription factor BIM2-like protein mRNA |
| 20 | FaBabeanB_3kb_HQ_transcript/5257 | transcription factor bHLH130 [Medicago truncatula] <AES67711.1 transcription factor bHLH122-like protein [Medicago truncatula] | V4KKA9.1 PF00010.23;HLH; | Medicago truncatula transcription factor bHLH122-like protein mRNA |
| 21 | FaBabeanB_3kb_HQ_transcript/5850 | hypothetical protein TSUD_322910 [Trifolium subterraneum] | A0A0D3DPE4.1 PF00010.23;HLH; | Medicago truncatula basic helix loop helix protein BHLH23 mRNA |
| 22 | FaBabeanB_3kb_HQ_transcript/6444 | transcription factor PIF4 isoform X2 [Medicago truncatula] | M0STT9.1 PF00010.23;HLH; | Medicago truncatula transcription factor partial mRNA |
| 23 | FaBabeanB_3kb_HQ_transcript/6602 | hypothetical protein TSUD_172970 [Trifolium subterraneum] | B9IBW9.2 PF00010.23;HLH; | PREDICTED: Cicer arietinum transcription factor UNE10 (LOC101492173), mRNA |
| 24 | FaBabeanB_3kb_HQ_transcript/6688 | hypothetical protein TSUD_109710 [Trifolium subterraneum] | V4KKA9.1 PF00010.23;HLH; | Medicago truncatula transcription factor bHLH122-like protein mRNA |
| 25 | FaBabeanB_3kb_HQ_transcript/7482 | transcription factor PIF1 isoform X1 [Medicago truncatula] <XP_024625778.1 transcription factor PIF1 isoform X1 [Medicago truncatula] <AES81693.1 transcription factor [Medicago truncatula] | A0A022PNX7.1 PF00010.23;HLH; | Medicago truncatula transcription factor mRNA |
| 26 | FaBabeanB_3kb_HQ_transcript/7624 | hypothetical protein TSUD_172970 [Trifolium subterraneum] | B9IBW9.2 PF00010.23;HLH; | PREDICTED: Cicer arietinum transcription factor UNE10 (LOC101492173), mRNA |
| 27 | FaBabeanB_3kb_HQ_transcript/8089 | transcription factor bHLH144-like protein [Trifolium pratense] | M1AF13.1 PF00010.23;HLH; | PREDICTED: Cicer arietinum transcription factor bHLH144-like (LOC101491896), mRNA |
| 28 | FaBabeanB_3kb_HQ_transcript/8238 | transcription factor bHLH130 [Medicago truncatula] <AES67711.1 transcription factor bHLH122-like protein [Medicago truncatula] | V4KKA9.1 PF00010.23;HLH; | Medicago truncatula transcription factor bHLH122-like protein mRNA |
| 29 | FaBabeanB_3kb_HQ_transcript/8552 | hypothetical protein TSUD_333600 [Trifolium subterraneum] | G7ICN0.1 PF00010.23;HLH; | PREDICTED: Cicer arietinum transcription factor UNE12 (LOC101502533), transcript variant X2, mRNA |
| 30 | FaBabeanB_3kb_HQ_transcript/8643 | hypothetical protein TSUD_413200 [Trifolium subterraneum] | V4KKA9.1 PF00010.23;HLH; | Medicago truncatula transcription factor bHLH122-like protein mRNA |
| 31 | FaBabeanB_3kb_HQ_transcript/8747 | hypothetical protein TSUD_172970 [Trifolium subterraneum] | B9IBW9.2 PF00010.23;HLH; | PREDICTED: Cicer arietinum transcription factor UNE10 (LOC101492173), mRNA |
| 32 | FaBabeanB_3kb_HQ_transcript/8850 | hypothetical protein TSUD_172970 [Trifolium subterraneum] | B9IBW9.2 PF00010.23;HLH; | PREDICTED: Cicer arietinum transcription factor UNE10 (LOC101492173), |

| | | | | |
|---|---|---|---|---|
| | | | | mRNA |
| 33 | FaBabeanB_3kb_HQ_transcript/8965 | transcription factor PIF1-like protein, partial [Trifolium pratense] | A0A022PNX7.1 PF00010.23;HLH; | Medicago truncatula transcription factor mRNA |
| 34 | FaBabeanB_3kb_HQ_transcript/9166 | PREDICTED: transcription factor bHLH48 [Cicer arietinum] | K3XXX3.1 PF00010.23;HLH; | PREDICTED: Cicer arietinum transcription factor bHLH48 (LOC101495752), mRNA |
| 35 | FaBabeanB_3kb_HQ_transcript/9563 | transcription factor UNE12 [Medicago truncatula] <KEH43018.1 transcription factor UNE12-like protein [Medicago truncatula] | G7ICN0.1 PF00010.23;HLH; | Medicago truncatula transcription factor UNE12-like protein mRNA |
| 36 | FaBabeanB_3kb_HQ_transcript/9973 | hypothetical protein TSUD_26280 [Trifolium subterraneum] | G7ICN0.1 PF00010.23;HLH; | PREDICTED: Cicer arietinum transcription factor UNE12-like (LOC101488285), mRNA |
| 37 | FaBabeanB_3kbup_HQ_transcript/15686 | hypothetical protein TSUD_208340 [Trifolium subterraneum] | M0TJ47.1 PF00010.23;HLH; | Medicago truncatula transcription factor BIM2-like protein mRNA |
| 38 | FaBabeanB_3kbup_HQ_transcript/15902 | hypothetical protein TSUD_388230 [Trifolium subterraneum] | I1GN25.1 PF00010.23;HLH; | Medicago truncatula BHLH transcription factor-like protein mRNA |
| 39 | FaBabeanB_3kbup_HQ_transcript/17060 | phytochrome-interacting factor 3.1 [Medicago truncatula] | M0YQ17.1 PF00010.23;HLH; | Medicago truncatula phytochrome-interacting factor 3.1 mRNA |
| 40 | FaBabeanB_3kbup_HQ_transcript/17409 | hypothetical protein TSUD_172970 [Trifolium subterraneum] | B9IBW9.2 PF00010.23;HLH; | PREDICTED: Cicer arietinum transcription factor UNE10 (LOC101492173), mRNA |
| 41 | FaBabeanB_3kbup_HQ_transcript/19322 | hypothetical protein TSUD_172970 [Trifolium subterraneum] | B9IBW9.2 PF00010.23;HLH; | PREDICTED: Cicer arietinum transcription factor UNE10 (LOC101492173), mRNA |
| 42 | FaBabeanB_3kbup_HQ_transcript/21105 | PREDICTED: transcription factor bHLH79-like [Cicer arietinum] | K7K628.1 PF00010.23;HLH; | Medicago truncatula chromosome 5 clone mth4-7g23, COMPLETE SEQUENCE |
| 43 | FaBabeanB_3kbup_HQ_transcript/21273 | PREDICTED: transcription factor PIF3 isoform X2 [Cicer arietinum] | A0A0D3EMM9.1 PF00010.23;HLH; | Medicago truncatula phytochrome-interacting factor 3.1 mRNA |
| 44 | FaBabeanB_3kbup_HQ_transcript/23879 | PREDICTED: transcription factor PIF3 isoform X2 [Cicer arietinum] | A0A0D3EMM9.1 PF00010.23;HLH; | Medicago truncatula phytochrome-interacting factor 3.1 mRNA |
| 45 | FaBabeanB_3kbup_HQ_transcript/24101 | transcription factor PIF4 isoform X1 [Medicago truncatula] <KEH33641.1 transcription factor [Medicago truncatula] | M0STT9.1 PF00010.23;HLH; | Medicago truncatula transcription factor partial mRNA |

e) List of COR genes selected from the Fababean full-length gene set.

| No. | Contig | NR | Pfam | NT |
|---|---|---|---|---|
| 1 | FaBabeanB_3kb_HQ_transcript/14478 | chlorophyll a-b binding protein CP24 10A, chloroplastic [Medicago truncatula] | PF00504 | Medicago truncatula light-harvesting complex I chlorophyll A/B-binding protein mRNA |
| 2 | FaBabeanB_3kb_HQ_transcript/15109 | chlorophyll a-b binding protein CP24 10A, chloroplastic [Medicago truncatula] | PF00504 | Medicago truncatula light-harvesting complex I chlorophyll A/B-binding protein mRNA |
| 3 | FaBabeanB_3kb_HQ_transcript/16562 | chlorophyll a-b binding protein CP24 10A, chloroplastic [Medicago truncatula] | PF00504 | Medicago truncatula light-harvesting complex I chlorophyll A/B-binding protein mRNA |
| 4 | FaBabeanB_3kb_HQ_transcript/17484 | Chain 4, Structure Of Stacked C2s2m2-type Psii-lhcii Supercomplex From Pisum Sativum | PF00504 | Medicago truncatula light-harvesting complex I chlorophyll A/B-binding protein mRNA |
| 5 | FaBabeanB_3kb_HQ_transcript/18387 | Chain 4, Structure Of Stacked C2s2m2-type Psii-lhcii Supercomplex From Pisum Sativum | PF00504 | Medicago truncatula light-harvesting complex I chlorophyll A/B-binding protein mRNA |
| 6 | FaBabeanB_3kb_HQ_transcript/18974 | Chain 4, Structure Of Stacked C2s2m2-type Psii-lhcii Supercomplex From Pisum Sativum | PF00504 | Medicago truncatula light-harvesting complex I chlorophyll A/B-binding protein mRNA |
| 7 | FaBabeanB_3kb_HQ_transcript/19075 | Chain 4, Structure Of Stacked C2s2m2-type Psii-lhcii Supercomplex From Pisum Sativum | PF00504 | Medicago truncatula light-harvesting complex I chlorophyll A/B-binding protein mRNA |
| 8 | FaBabeanB_3kb_HQ_transcript/19425 | PREDICTED: chlorophyll a-b binding protein CP24 10A, chloroplastic [Cicer arietinum] | PF00504 | Medicago truncatula light-harvesting complex I chlorophyll A/B-binding protein mRNA |
| 9 | FaBabeanB_3kb_HQ_transcript/19606 | PREDICTED: chlorophyll a-b binding protein CP24 10A, chloroplastic [Cicer arietinum] | PF00504 | Medicago truncatula light-harvesting complex I chlorophyll A/B-binding protein mRNA |

f) List of DHN2 genes selected from the Fababean full-length gene set.

| No. | Contig | NR | Pfam | NT |
|---|---|---|---|---|
| 1 | FaBabeanB_3kb_HQ_transcript/15951 | HVA22-like protein a isoform X1 [Medicago truncatula] | PF03134 | *Medicago truncatula* TB2/DP1, HVA22 family protein mRNA |

Supplementary Table 6. Primer information used for expression analysis

| Primer Name | Primer sequences (5′ →3′) |
|---|---|
| WCOR413_15785 | (F) TGT GCA ACA AGA AAG CAA GC |
| | (R) CAG TTC GTG CAT TTG CAG TT |
| DHN2_12403 | (F) CCA CTC ATC AAT ATC GGT CAC |
| | (R) TTG ACT ATA CAT GCA CAC ATC AAA A |
| DHN2_14197 | (F) GGC CTT GTG TGG TGA TTT CT |
| | (R) GAA CCA AAC ACA TAT CAT CAA GA |
| DHN2_14797 | (F) GCG CTC TCT CAT CTC TCA GC |
| | (R) CTG ATT CTC CTC AGC CAT GAT |
| COR15_14478 | (F) TGG ACA AGG GAA GAC TCC AC |
| | (R) CAG ACA TAA GGT AAA TGC ATC CAA |

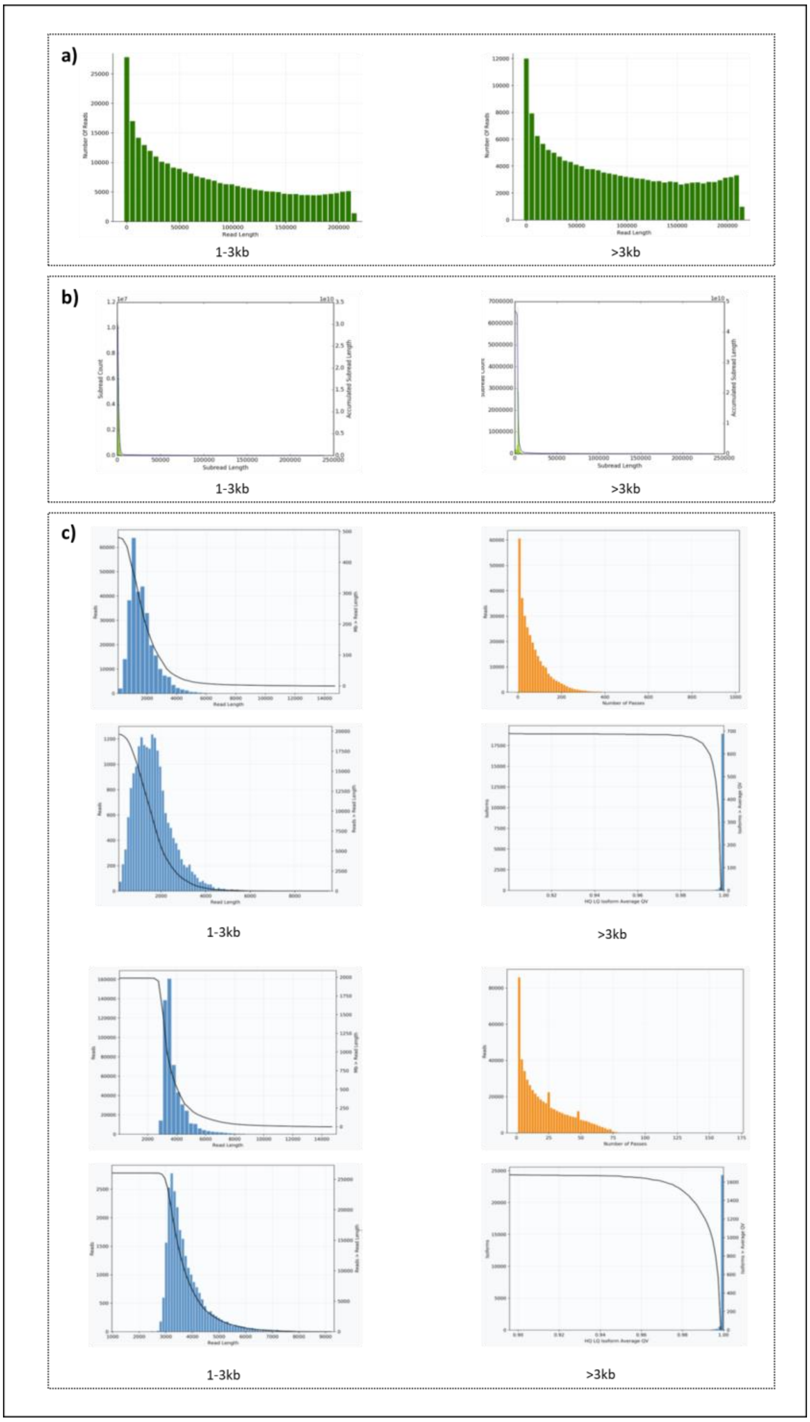


Supplementary Figure 1. Summary of a) Polymerase read length distribution b) Subread length distribution c) CCS read length distribution; Number of passes; Read length of consensus isoform reads; Average Quality value of HQ and LQ isoforms

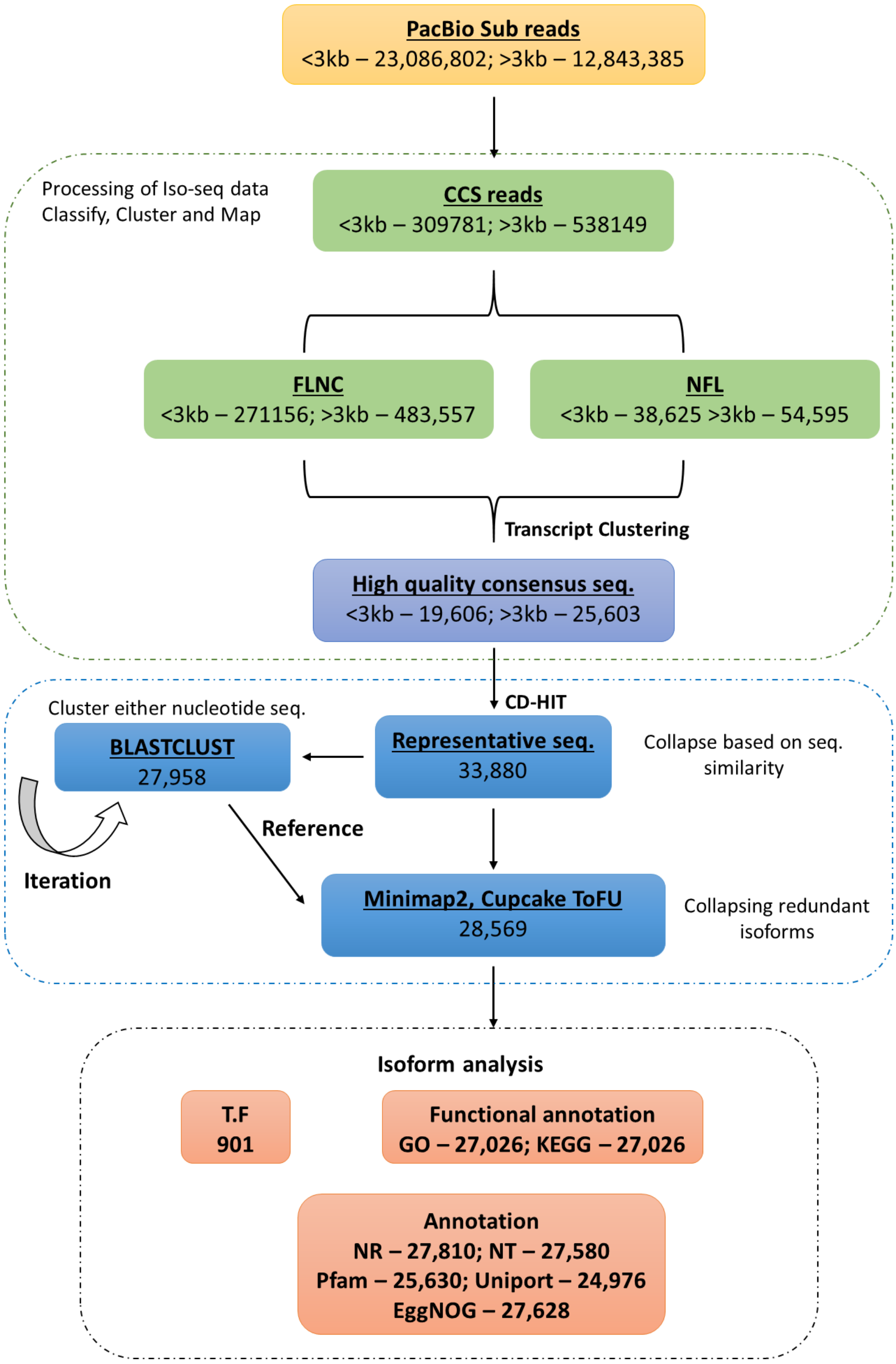


Supplementary Figure 2. Basic Iso-seq workflow
